# Just a Bad Case of Cotton Fever: A Case Report and Literature Review

**DOI:** 10.7759/cureus.28352

**Published:** 2022-08-24

**Authors:** Hanisha Geedipally, Sabin Karki, Saloni Shirke, Ronak Bhimani

**Affiliations:** 1 Internal Medicine, Lower Bucks Hospital, Bristol, USA; 2 Medicine, Caribbean Medical University School of Medicine, Willemstad, CUW; 3 Hospital Medicine, Lower Bucks Hospital, Bristol, USA

**Keywords:** fever of unkown origin, endocarditis, sepsis, opiods, iv drugs, cotton fever

## Abstract

Fever is one of the most commonly seen presentations in intravenous drug abusers. "Cotton Fever" is a benign condition, unrecognized among the medical community. It is characterized by a systemic inflammatory response syndrome occurring within minutes of intravenous (IV) drug use. Patients present to tertiary-level care settings with fever, chills, gastrointestinal symptoms, shortness of breath, and chest pain. We present the case of a 46-year-old Caucasian male who presented with lightheadedness, chest pain, and gastrointestinal symptoms after using IV methamphetamine. On physical examination, he was disoriented, tachycardic, and had a fever of 102.8⁰F. He did not have any Osler nodes, Janeway lesions, or splinter hemorrhages. Diagnostics showed leukopenia with neutropenia, lactic acidosis, and elevated creatine kinase. Blood cultures did not grow any organisms. The patient was admitted to the intensive care unit and treated with IV fluids and broad-spectrum antibiotics. His condition improved rapidly and the patient left against medical advice (AMA).

The toxic appearance of patients presenting with cotton fever often causes panic among clinicians, resulting in an extensive diagnostic workup, exhaustion of hospital resources, and overprescription of antibiotics. Due to the lack of knowledge about this condition among healthcare practitioners and the tendency of these patients to leave AMA, an appropriate management strategy remains unrecognized. Our case aims to bring awareness about this condition to help guide patient-directed care, reduce the use of healthcare resources, and establish prevention strategies in the community.

## Introduction

In the year 2020, 5.7 million people aged 12 or older in the United States used cocaine (0.58%), methamphetamines (0.26%), and heroin (0.16%) [1]. People who inject drugs (PWID) make up a large proportion of this cohort. In this population, fever is one of the most commonly seen presentations. The list of differential diagnoses is lengthy. Among these is a rare entity, unrecognized in the medical community but well known among PWIDs: “Cotton Fever”. This ailment results when intravenous drug abusers (IVDA) re-inject trace amounts of drugs extracted from a previously used cotton filter. In addition to fever, other symptoms include rigors, headache, nausea, vomiting, and myalgia. This may be mistaken for a sepsis-like clinical presentation, resulting in a redundant diagnostic workup, overuse of hospital resources, and prescription of unnecessary treatments [2-15]. We aim to raise more awareness about this disease in the healthcare community by presenting a case of cotton fever in a patient with recent intravenous (IV) methamphetamine use. 

## Case presentation

Minutes after IV methamphetamine use, a 46-year-old Caucasian male presented to the emergency department (ED) with complaints of lightheadedness, nausea, vomiting, abdominal cramping, and intermittent chest pain. The history was limited because of the patient's mental status (the patient was given a dose of IV Ativan in the ED for restlessness and erratic behavior). He reported experiencing similar episodes in the past but the current symptoms were longer in duration, which led him to seek care at our facility. The patient attributed his symptoms to a “bad batch” and stated, it was “just a bad case of cotton fever”. 

His past medical history was significant for methicillin-resistant *Staphylococcus* cellulitis of his arm (requiring extensive debridement) and an ongoing poison-ivy dermatitis on his right lower extremity. His family history was non-contributory. He was a current daily smoker (0.5 packs per day) and reported injecting 20 mg of methamphetamines every day. He also admitted to using marijuana, fentanyl, and heroin, but denied current alcohol use. 

Physical examination showed a disheveled and unkempt Caucasian male, in no acute distress. He was somnolent and oriented to place but not time and person. He was able to respond to questions. Cardiovascular examination was significant for tachycardia but regular rhythm, normal S1, and S2 heart sounds, and no murmurs, rubs, or gallops. Examination of the skin showed a vesicular rash on the right proximal calf without signs of an abscess or cellulitis. Osler nodes, Janeway lesions, or splinter hemorrhages were not observed. Pulmonary, abdominal, musculoskeletal, and neurological examinations were all within normal limits. His temperature was 102.8⁰F, respiratory rate (RR) was 28 per minute, blood pressure was 111/55, pulse was 123 per minute and oxygen saturation was 96% on room air. 

**Figure 1:**
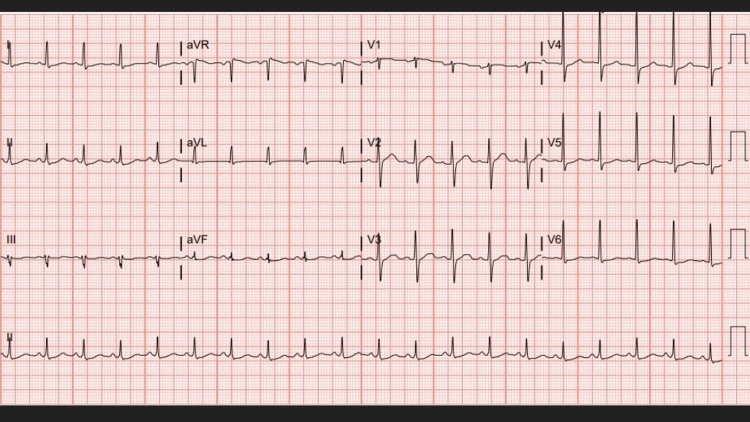
Patient's electrocardiogram on admission

**Figure 2:**
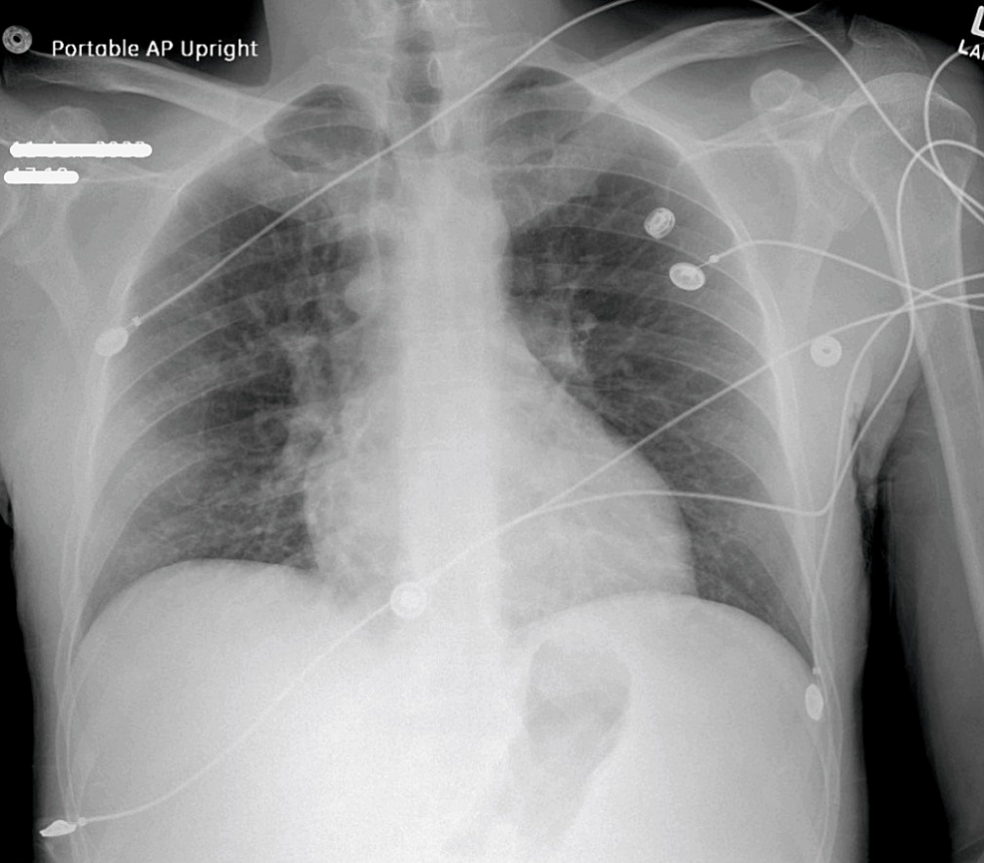
Patient's chest x-ray on admission

On admission, lab findings were significant for lactic acidosis, leukopenia, neutropenia, and elevated creatine kinase (CK) (Tables 1, 2). A urine drug panel was positive for amphetamines. Urinalysis findings were consistent with myoglobinuria (Table 3). An electrocardiogram (ECG) showed regular sinus rhythm (Figure 1) and troponins (<0.01 ng/mL) were negative. A chest x-ray was unremarkable (Figure 2). The patient was admitted to the intensive care unit (ICU) for sepsis with a neutropenic fever and methamphetamine overdose. 

**Table 1:** Patient's complete blood count on admission WBC: white blood cell count; RBC: red blood cells; Hgb: hemoglobin; Hct: hematocrit; Plt: platelets

Complete Blood Count	
WBC	1.7 x 10³ uL
Neutrophils	1.4 x 10³ uL
Lymphocytes	0 x 10³ uL
Monocytes	0.1 x 10³ uL
Basophils	0 x 10³ uL
RBC	4.2 x 10⁶ uL
Hgb	11.6 g/dL
Hct	34.40%
Plt	205 x 10³ uL

**Table 2:** Patient's serum chemistry on admission BUN: blood urea nitrogen; AST: aspartate aminotransferase; ALT: alanine aminotransferase; ALP: alkaline phosphatase

Serum Chemistry	
Sodium	140 mmol/L
Potassium	3.0 mmol/L
Chloride	104 mmol/L
Bicarbonate	21.6 mmol/L
BUN	21 mmol/L
Creatinine	1.1 mmol/L
Glucose	100 mg/dL
AST	54 U/L
ALT	46 U/L
ALP	111 U/L
Albumin	3.6 g/dL
Total bilirubin	4.2 mg/dL
Lipase	117 U/L
Calcium	8.4 mg/dL
Lactate	3.6 mmol/L
Creatine kinase	500 U/L

**Table 3:** Patient's urinalysis RBCs: red blood cells; WBCs: white blood cells

Urinalysis	
Specific gravity	1.025
Color	Dark yellow
Clarity	Clear
pH	6.0
Leukocyte esterases	Negative
Nitrites	Negative
Protein	Trace
Glucose	Negative
Ketones	Negative
Urobilinogen	Negative
Bilirubin	1+
Blood	1+
RBCs	Negative
WBCs	Negative

Treatment was started with IV normal saline, vancomycin, piperacillin-tazobactam, electrolyte repletion, and as-needed acetaminophen, as well as diphenhydramine and calamine ointment (for contact dermatitis). Infectious disease was consulted. Eight hours after hospitalization, the patient’s leukocyte count increased to 6.5 x 10³ uL and absolute neutrophil count to 4.7 x 10³ uL. On follow-up 24 hours after admission, the patient showed significant clinical improvement, with resolving tachycardia. The lactic acid levels normalized and CK levels began trending down. Blood cultures on admission did not grow any organisms. We planned to downgrade the patient to the medical floors, de-escalate antibiotics, and obtain a transthoracic echocardiogram to rule out infective endocarditis (given his risk factors). However, the patient decided to leave against medical advice (AMA) and was unfortunately lost to follow-up. Repeat blood cultures 24 hours after admission were negative. A diagnosis of cotton fever was established given the clinical presentation, lack of evidence supporting other diagnoses, and a benign, self-limiting course of the illness.

## Discussion

Fever is a common presentation among IVDA. Differential diagnoses commonly include infectious causes such as infective endocarditis (IE), community-acquired pneumonia, aspiration pneumonia, active tuberculosis, skin and soft tissue infections, bacteremia, fungemia, viral infections (hepatitis B and C, HIV), clostridial infections, and (rarely) anthrax [16]. Less commonly, other non-infectious etiologies include vasculitis, sympathomimetic effects of stimulant drugs, rhabdomyolysis, and cannabinoid hyperemesis syndrome [17].

Initially described by Thomson et al. in 1975, cotton fever (or “shooting the cottons”) is an underrecognized cause of fever among medical practitioners [18]. PWIDs use cotton, membrane, or cigarette filters to remove insoluble particles from drug preparations. The retained particles are often stored and re-injected after extraction [19]. The intravenous administration of cotton contaminants causes a sepsis-like syndrome with transient, acute-onset fever, and leukocytosis [2,17]. 

To review the available literature on cotton fever, we used a systematic search strategy on PubMed using key terms or combinations such as "shooting the cotton", "fever", "cotton fever", "IV drugs", "fever and IV drugs", "infections and IV drug users", "fever and illicit drug use", and "febrile illness and IV drugs". We included relevant articles on cotton fever secondary to drug use. Studies related to pneumoconiosis from cotton exposure were excluded. As a result, we found only 13 case reports, one case series, and a single cross-sectional study (Table 4) [2-15].

**Table 4:** Summary of findings from the available literature on cotton fever IV: intravenous; WBC: white blood cell; AMA: against medical advice; PMNs: polymorphonuclear cells; TEE: transesophageal echocardiogram

Author (Year)	Study Design	Age	Ethnicity	Gender	IV Drug	Presenting Symptoms	Leukocyte & Neutrophil Count	Bacteremia	Endocarditis	Treatment	Prognosis
Shragg et al. (1978) [3]	Case Series	Case 1: 23 Case 2: 22	Not reported	Case 1: Female Case 2: Male	Case 1: Heroin Case 2: Heroin	Case 1: Acute onset of shaking chills, nausea, diffuse abdominal pains, intense muscular aches, fever Case 2: back, leg, and abdominal pains, headache, nausea, vomiting, shortness of breath, fever	Case 1: WBC: 21700 mm^3^ (neutrophils: 29%) Case 2: WBC: 5800 mm^3^	Case 1: Absent Case 2: Absent	No reported evidence in both cases	Case 1: Began therapy for endocarditis, Observation Case 2: Observation	Case 1: Patient left AMA shortly after arrival on the ward Case 2: Persistent leukocytosis which was followed up outpatient, after 2 days the patient's WBC count normalized
Wright et al. (1980) [4]	Case Report (Abstract)	24	African American	Female	Talwin	Fever, nausea, vomiting, shaking, chills, headaches, myalgias, polyarthralgia, severe abdominal pain, and muscle contractions	Not reported	Absent	Ruled out	Not reported	Not reported
Harrison et al. (1990) [5]	Case Report	33	Not reported	Female	Talwin and Methylphenidate	Sudden onset of malaise, chills, abdominal and leg pain	WBC: 4200/mm^3^ PMNs: 57% Bands: 32% Lymphocytes: 11%	Absent	Ruled out	Intravenous fluids, thiamine, and observation for 48 hours	Resolved after 48 hours and discharged with no further follow-up available
Ferguson et al. (1993) [6]	Case Report	28	Not reported	Male	Heroin	Fever, chills, shortness of breath, lethargy, inattentive, diaphoretic	2.79 x 10^9^/L Neutrophils: 62% Repeat 24 x 10^9^/L Neutrophils: 91%	Enterobacter agglomerans	No evidence of endocarditis	Vancomycin and gentamicin; switched to cefazolin, then TMP-SMX	Resolved with 14 days of treatment, follow-up leukocytosis resolved
Ramik et al. (2008) [7]	Case Report	36	Caucasian	Male	Dilaudid	Fever, headache, myalgias, muscle spasms of the upper and lower extremities, nausea, vomiting, and rigors	None reported	Cultures not obtained	Ruled out	Not reported	Not reported
Torka et al. (2013) [8]	Case Report	22	Not reported	Male	Heroin	Diaphoresis, fever	9700 u/L	Absent	Absent	Ceftriaxone and Vancomycin, Intravenous fluid resuscitation	Fever resolved within 12-h of presentation, discharged in 48 hours, and stated intention to follow-up outpatient for detoxification
Ramirez et al. (2014) [9]	Case Report (Abstract)	19	Not Reported	Female	Heroin	Fever, chills, abdominal pain, vomiting, weakness, diaphoresis	WBC: 34000 Neutrophils: 69% Bands: 29%	Absent	Ruled out	Vancomycin, piperacillin-tazobactam, fluid resuscitation	Resolved within 8 hours of admission
Gugelmann et al. (2015) [10]	Case Report (Abstract)	37	Not Reported	Male	Methamphetamine	Dysphoria, shaking chills, angor animi	WBC: increased from 6000/uL to 21000/uL (94% neutrophil)	*Propionibacterium acnes* (contaminant)	Ruled out	Broad-spectrum antibiotics, 7-L of Normal Saline, Vasopressors (weaned over 9 hours after BP normalized)	Patient left AMA 14 hours after admission, and returned to the ER 4 days later because of the blood cultures, at the time he was asymptomatic, with normal labs and vital signs.
Holland et al. (2016) [11]	Case Report (Abstract)	24	Not reported	Female	Heroin	Severe headache, backache, and neck pain	WBC: 1400/mm^3^, repeat WBC: peaked 15700/mm3	Absent	No signs or indication	Vancomycin, cefepime	Transient with rapid improvement, discharged on day 2 with a presumed diagnosis of cotton fever
Xie et al. (2016) [12]	Case Report	22	Not reported	Female	Heroin	Fever, headache, abdominal pain, acute back pain	WBC: 22.6×10^9^/L with a left shift	Absent	Ruled out	Broad-spectrum antibiotics	"Brisk" clinical improvement within 24 hours
Zerr et al. (2016) [13]	Case Report	26	Not reported	Female	Heroin	Chills, fever, abdominal pain, chest pain, headache	WBC: 2000 k/mm^3^ Neutrophils: NR	Absent	Ruled out	Vancomycin, piperacillin-tazobactam	Afebrile within 4 hours of admission, left AMA
Burgin et al. (2017) [14]	Case Report (Abstract)	31	Not reported	Female	Not Specified	Dyspnea, chest pain, severe abdominal pain, right arm swelling, and generalized weakness	WBC: 13,000	Enterobacter agglomerans	Ruled out	Levofloxacin for 14 days	Not reported
Chandrika et al. (2019) [15]	Case Report (Abstract)	30	Not Reported	Male	Cocaine	Progressive fever, nausea, vomiting, chest pain, dyspnea	WBC: 34,000 with leftward shift	Absent	Ruled out	broad-spectrum antibiotics (discontinued after negative blood cultures), vasopressor support	Patient left AMA on the third day of hospitalization
Francis et al. (2019) [2]	Case Report	32	Caucasian	Male	Heroin	Fever, nausea, vomiting, palpitations	WBC: 4030/uL, Neutrophils 88.2%	Enterobacter asburiae	Mitral and pulmonic valve vegetations on TEE	Cefepime, ciprofloxacin	Left AMA

**Figure 3:**
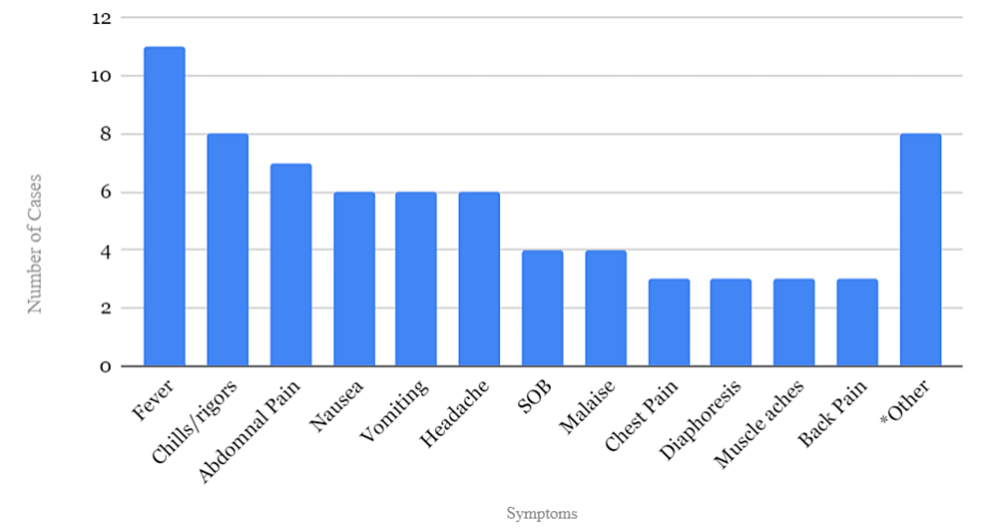
Most commonly reported symptoms of cotton fever SOB: shortness of breath *leg pain, polyarthralgia, palpitations, dysphoria, and angor animi

All cases reviewed were reported between 1975 and 2019. The average age of the patients was 27 years with a predominance of females (53%). Cotton fever was most closely associated with heroin use (60%), but pentazocine (also known as Talwin), methamphetamines, cocaine, and other opiates were also observed. The most commonly reported symptoms included fever, chills and rigors, abdominal pain, nausea, vomiting, shortness of breath, generalized weakness and malaise, chest pain, diaphoresis, myalgias, and back pain. Other symptoms such as leg pain, polyarthralgia, palpitations, dysphoria, and angor animi (defined as a fear of death) were less prevalent. Figure 3 summarizes our findings.

Our patient presented with lightheadedness, nausea, vomiting, abdominal cramping, and intermittent chest pain and met the systemic inflammatory response syndrome criteria. One of the mechanisms that can explain this pyrogenic response is the pharmacological theory, which hypothesizes that the water-soluble, heat-labile extracts from cotton are highly inflammatory. Alternatively, some experts believe that certain individuals have pre-formed antibodies to cotton, leading to a type II hypersensitivity response. However, evidence supporting both of these theories is lacking. The theory endorsed by most evidence hypothesizes that a gram-negative bacilli *Enterobacter agglomerans*, a prevalent colonizer in cotton, releases an endotoxin resulting in the sequelae of symptoms [12]. Contrarily, bacteremia with *E. agglomerans* is not present in affected patients but has been reported in rare cases [2,6,14].

Cotton fever is a benign, self-limited ailment; however, because PWIDs are at a high risk of developing serious infections, other causes of sepsis should be ruled out. Francis et al. reported a case of cotton fever and IE due to *Enterobacter asburiae*. Based on the modified Duke’s criteria for IE, we can rule out "definite IE" in our case; however, the pretest probability of "possible IE" is inconclusive because our patient left AMA before an echocardiogram was performed [20]. Nonetheless, IE was unlikely given the lack of positive blood cultures, cardiac murmurs on physical exam, or signs of immunologic and vascular phenomena. He also showed rapid resolution of fever and significant clinical improvement within 24 hours, which is less characteristic for IE but more common in cotton fever (Table 4) [5,9-14].

Additionally, rhabdomyolysis can also be considered as the etiology of our patient’s symptoms; however, the potassium levels were low and mild elevations in CK did not correlate with the patient’s toxic presentation. The patient's elevated CK levels and myoglobinuria may, in fact, be a result of amphetamine intoxication where the sympathomimetic effects can overlap with cotton fever. Other diagnoses including pneumonia, fungemia, and acute hepatitis or HIV were also unlikely given a normal chest x-ray, negative blood cultures, lack of significant elevations in liver function tests, and transient symptomatology, respectively. 

There are no established management guidelines for cotton fever. Most reported cases are treated with broad-spectrum antibiotics (Table 4), which carry adverse risks including *Clostridium difficile* infections. The use of conservative management with fluids and antipyretics may be sufficient [5,15]. Interestingly, most affected patients tend to leave AMA, making it difficult to determine the most effective treatment and understand the prognosis of the illness. Additional challenges may arise when trying to balance the use of appropriate medical resources (to rule out serious conditions) and treating a benign, transient illness. In our case, the patient’s neutropenic fever led to ICU admission and the use of antibiotic therapy. In retrospect, given our patient’s risk factors and toxic presentation, it is difficult to determine whether these were appropriate or inappropriate management choices. Some patients may present with clinical instability requiring vasopressors [10,15].

Nonetheless, less severe cases of cotton fever should be managed in short-term observational units after an appropriate risk versus benefit analysis [5]. This strategy may improve overall patient outcomes, provide better patient-centered care, decrease hospital costs, and limit unnecessary diagnostic studies. Furthermore, the incidence of cotton fever can be reduced through primary prevention by pharmacies and harm reduction facilities that supply cotton and membrane filters to PWIDs. IVDAs that use cotton filters are almost twice as likely to develop this condition [19]. Education about cotton fever and the increased availability of membrane filters can further help decrease the incidence of the illness and visits to the ED.

## Conclusions

Cotton fever is a benign, self-limiting illness that occurs in IVDAs, minutes after injecting drugs through filter preparations. Common symptoms include fever, chills, myalgia, malaise, shortness of breath, chest pain, and gastrointestinal symptoms. Diagnostic workup usually shows signs of a systemic inflammatory syndrome (leukocytosis or leukopenia, tachycardia, tachypnea). Risk stratification is a key element in the management of this condition. Patients will usually require IV fluids, antibiotics, and/or vasopressors after initial presentation. However, after negative diagnostic testing or in the setting of low suspicion for serious conditions such as infective endocarditis, skin infections, and rhabdomyolysis, a diagnosis of cotton fever should be suspected. Cotton fever can be treated conservatively, with a patient-centered approach. Patients with less severe symptoms can be managed under medical observation. Education about avoiding re-injection of IV drugs from reused filters can also help prevent the incidence of the condition and the risk of future hospitalizations. 

## References

[1] (2022). 2020 National Survey of Drug Use and Health (NSDUH) Releases. https://www.samhsa.gov/data/release/2020-national-survey-drug-use-and-health-nsduh-releases.

[2] Francis MJ, Chin J, Lomiguen CM, Glaser A (2020). Cotton fever resulting in Enterobacter asburiae endocarditis. IDCases.

[3] Shragg T (1978). "Cotton Fever" in narcotic addicts. JACEP.

[4] Wright J, Christopher CR (1980). Cotton fever and pregnancy. A confusing clinical problem. Diagn Gynecol Obstet.

[5] Harrison DW, Walls RM (1990). "Cotton fever": a benign febrile syndrome in intravenous drug abusers. J Emerg Med.

[6] Ferguson R, Feeney C, Chirurgi VA (1993). Enterobacter agglomerans-associated cotton fever. Arch Intern Med.

[7] Ramik D, Mishriki YY (2008). The other "cotton fever". Infect Dis Clin Pract (Baltim Md).

[8] Torka P, Gill S (2013). Cotton fever: an evanescent process mimicking sepsis in an intravenous drug abuser. J Emerg Med.

[9] Ramirez MD, Marsh B (2014). Cotton fever: a self-limiting syndrome in IVDA. J Investig Med.

[10] Gugelmann HM, Durrani T (2015). Bong water cotton fever: parenteral administration of sterilized, desiccated and reconstituted methamphetamine water pipe runoff [Abstract]. Clin Toxicol.

[11] Holland R, Franasiak R, Wittler M (2016). 1740: Cotton fever: the great imitator. Crit Care Med.

[12] Xie Y, Pope BA, Hunter AJ (2016). Cotton fever: does the patient know best?. J Gen Intern Med.

[13] Zerr AM, Ku K, Kara A (2016). Cotton fever: a condition self-diagnosed by IV drug users. J Am Board Fam Med.

[14] Burgin A, Lockwood W, Mastalerz K (2022). Use clean needles, boil your cotton: advice for the modern drug user [Abstract]. 2017 ACP Colorado Chapter Meeting.

[15] Chandrika P, Hussain J, Ghadermarzi S (2019). Early to pressors, early to leave against medical advice-cotton fever in IV drug user. Am J Respir Crit Care Med.

[16] Lavender TW, McCarron B (2013). Acute infections in intravenous drug users. Clin Med (Lond).

[17] Wurcel AG, Merchant EA, Clark RP, Stone DR (2015). Emerging and underrecognized complications of illicit drug use. Clin Infect Dis.

[18] Thomson BD (1975). Medical complications following intravenous heroin. Ariz Med.

[19] Mezaache S, Briand-Madrid L, Laporte V (2020). Correlates of self-reported cotton fever experience among people who inject opioids. Subst Use Misuse.

[20] Durack DT, Lukes AS, Bright DK (1994). New criteria for diagnosis of infective endocarditis: utilization of specific echocardiographic findings. Duke endocarditis service. Am J Med.

